# Oligo(ethylene glycol)-sidechain microgels prepared in absence of cross-linking agent: Polymerization, characterization and variation of particle deformability

**DOI:** 10.1371/journal.pone.0181369

**Published:** 2017-07-18

**Authors:** Nicole Welsch, L. Andrew Lyon

**Affiliations:** 1 School of Chemistry and Biochemistry, Georgia Institute of Technology, Atlanta, GA, United States of America; 2 The Parker H. Petit Institute for Bioengineering and Biosciences, Georgia Institute of Technology, Atlanta, GA, United States of America; 3 Schmid College of Science and Technology, Chapman University, Orange, CA, United States of America; Martin-Luther-Universitat Halle-Wittenberg, GERMANY

## Abstract

We present a systematic study of self-cross-linked microgels formed by precipitation polymerization of oligo ethylene glycol methacrylates. The cross-linking density of these microgels and, thus, the network flexibility can be easily tuned through the modulation of the reaction temperature during polymerization. Microgels prepared in absence of any difunctional monomer, i.e. cross-linker, show enhanced deformability and particle spreading on solid surfaces as compared to microgels cross-linked with varying amounts of poly(ethylene glycol diacrylate) (PEG-DA) in addition to self-crosslinking. Particles prepared at low reaction temperatures exhibit the highest degree of spreading due to the lightly cross-linked and flexible polymer network. Moreover, AFM force spectroscopy studies suggest that cross-linker-free microgels constitute of a more homogeneous polymer network than PEG-DA cross-linked particles and have elastic moduli at the particle apex that are ~5 times smaller than the moduli of 5 mol-% PEG-DA cross-linked microgels. Resistive pulse sensing experiments demonstrate that microgels prepared at 75 and 80°C without PEG-DA are able to deform significantly to pass through nanopores that are smaller than the microgel size. Additionally, we found that polymer network flexibility of microgels is a useful tool to control the formation of particle dewetting patterns. This offers a promising new avenue for build-up of 2D self-assembled particle structures with patterned chemical and mechanical properties.

## Introduction

Microgels are hydrated, cross-linked polymer networks with sizes typically in the colloidal range and, in contrast to solid colloidal particles, are mechanically soft materials. In addition, they display complex, stimuli triggered swelling/deswelling behavior as consequence of their characteristic network structure and polymer composition.[1, 2] By virtue of their soft character and the possibility to vary their mechanical and chemical properties, microgels continue to be heavily investigated as viable materials for many biological applications. For example, microgel particles have been designed as drug delivery vehicles[3–5], enzyme supports[6, 7], biosensors[8, 9], and hemostatic agents[10]. Recently, microgels have also been used with great success as building blocks in a bottom-up approach to develop responsive surface coatings[11–13], self-healing materials[14], and tissue engineering scaffolds[15, 16].

The properties of microgels including size, colloidal stability, swelling, environmental responsivity, and stiffness can be tailored through the modulation of the reaction conditions as well as the choice and concentration of monomer, co-monomer(s) and cross-linking agent. Herein, the concentration of cross-links and the distribution of cross-linking points are the most important parameters which influence the mechanical properties of the microgel particle, such as particle spreading on surfaces and deformability. The most widely studied thermo-responsive microgels are those composed of poly(*N*-isopropylacrylamide) (pNiPAm), which are traditionally prepared by precipitation polymerization from *N*-isopropylacrylamide (NiPAm) in presence of a cross-linking agent, such as *N*,*N*´methylene-bisacrylamide (BIS). Below 32°C, *i*.*e*., the lower critical solution temperature (LCST) of pNiPAm[17], the elastic modulus of pNiPAm microgels can be adjusted in a range between tens and hundreds of kPa depending on the concentration of BIS, and thus the degree of cross-linking.[18]

The groups of Pelton and Frisken were the first to demonstrate that it is also possible to prepare pNiPAm microgels in absence of a di-functional monomer as cross-linking agent.[19–21] The formation of three-dimensional polymer networks in absence of bifunctional molecules was attributed to chain transfer reactions *via* tert-C atoms at the backbone or the sidechain of the growing pNiPAm polymers. Considering the stability and kinetics of mid-chain radical formation[22], chain transfer reactions are rare resulting in ultra-low cross-linked (~0.25 mol-%) polymer networks.[21] Due to the low connectivity of polymer chains within the formed network, cross-linker-free pNiPAm microgels show high levels of deformability and spreading.[23] The mechanical behavior of soft hydrogels has become an important research area for various applications including biomaterial design.[24] For example, Merkel *et al*. designed hydrogel microparticles of ellipsoidal shape and demonstrated that particle deformability governs the *in vivo* blood circulation times as well as biodistribution.[25] Moreover, it has been recently shown in our group that highly deformable ultra-low cross-linked microgels conjugated to fibrin-binding motifs are superior in binding and contracting fibrin matrices compared to stiffer BIS-cross-linked pNiPAm microgels, making these constructs potential agents to reduce bleeding in case of an injury or in patients with certain deficiencies in clotting pathways such as those common with neonatal cardiopulmonary bypass patients.[10]

Formation of gels during free radical polymerization *via* chain transfer reactions is not limited to pNiPAm hydrogels, but has been observed using other monomers, such as *N*-vinylformamide and *N*,*N*´-dimethylacrylamide.[26, 27] Herein, we report on the preparation and characterization of cross-linker-free microgels from poly(ethylene glycol) (PEG) side-chain monomers. These microgel particles can be synthesized by precipitation polymerization of poly/oligo ethylene glycol (meth)acrylates (OEG(M)A). In case of OEG(M)A-based polymers the LCST varies depending on the polymerization degree of the ethylene oxide side chains.[28–31] PEGs are generally considered to have a low toxicity, good biocompatibility and anti-fouling behavior and have been shown to be safe using a large range of administration routes and molecular weights. For this reason PEGs are widely used in many products of daily life, e.g. tooth paste, and as excipient in many biomedical applications[32, 33] Several studies demonstrate that non-linear polymers constructed from monomers carrying oligo(ethylene glycol) sidechains, such as POEGMA, display similar bio-inert and non-cytotoxic properties when compared to PEG which explains the growing interest for these materials from the biomedical field.[28, 29, 34–38] To the best of our knowledge, long-term *in vivo* studies of OEGMA-based polymers and their degradation products remain to be carried out to fully evaluate their biocompatibility. In our study we used oligo ethylene glycol methacrylate containing 4–5 ethylene oxide units at its side chain (OEGMA_300_, *M*_n_ ~ 300 g/mol) as the main monomer. Since pOEGMA_300_ exhibits a LCST of about 64°C[28], the resulting microgel particles are hydrated and swollen at physiological conditions. On the basis of reaction kinetics, we hypothesized that the reaction temperature would be the key parameter to control cross-linking densities. We prepared p(OEGMA_300_-*co*-MAA) particles in absence of any difunctional monomer at different temperatures and compared their mechanical properties, such as swelling and deformation behavior after surface deposition as well as elastic moduli using atom force microscopy (AFM). Microgels containing different amounts of cross-linker poly(ethylene glycol diacrylate) (PEG-DA) were synthesized for comparison. To further characterize structural differences of the polymer networks in solely self-cross-linked and PEG-DA cross-linked microgels, we spatially analyzed the elastic modulus across the particle cross-section. In general, microgels prepared by precipitation polymerization in the presence of cross-linking agent have been found to exhibit significant structural heterogeneities. One reason for this finding is the mismatched reactivity ratios between cross-linking agent and main monomer.[39–43] In consequence of three-dimensional network inhomogeneities, hydrogel particles polymerized in presence of bifunctional molecules are characterized by pronounced stiffness gradients across the particle cross-section.[11, 18] The spatial analysis of the elastic properties of microgels prepared without difunctional monomers will give first insights into the chemical structure of these particles. In the second part of this paper, we reinforce the fact that microgel mechanics govern many different processes, such as nanopore translocation, surface spreading, and patterned self-assembly.

## Experimental section

### Materials

Chemicals were purchased from Sigma-Aldrich unless otherwise specified. Olig(othylene glycol) methyl ether methacrylate (*M*_n_ = 300, OEGMA_300_) and poly(ethylene glycol) (200) diacrylate (PEG-DA, Polysciences Inc.) were passed through a column of basic Al_2_O_3_ to remove the inhibitor prior to polymerization. Methacrylic acid (MAA), methacryloxyethyl thiocarbomoyl rhodamine B (Poly Fluor 570, Polysciences Inc.), potassium persulfate (KPS), and (3-aminopropyl)-trimethoxysilane (APTMS) were used as received. The following reagents were used to prepare buffers: 4-(2-hydroxyethyl)-1-piperazineethanesulfonic acid (HEPES), sodium chloride (BDH Chemicals), and sodium hydroxide. Acetone (BDH Chemicals), isopropanol (BDH Chemicals), and ethanol (VWR) were used as received. All water was distilled and deionized (Barnstead E-Pure) to a resistance of 18 MΩ. Additional particulate matter was removed using a 0.2 *μ*m filter.

### Particle synthesis

All microgels were synthesized *via* surfactant-free precipitation polymerization in a 250 mL three-necked round bottom flask equipped with a reflux condenser, N_2_ inlet, thermometer, and magnetic stirrer. OEGMA_300_ (88, 93, 97, or 98 mol-%; 72.5, 76.6, 79.9, or 80.7 mM final concentration) and PEG-DA, (0, 1, 5, or 10 mol-%; 0, 0.8, 4.1, or 8.2 mM final concentration) were dissolved into 84 mL DI H_2_O and the solution was degassed at room temperature by flushing with nitrogen for 20 min while stirring. In the next step, the solution was heated to 80°C in an oil bath and purged with nitrogen while stirring at 450 rpm. MAA (2 mol-%; 1.6 mM final concentration) dissolved in 0.5 mL DI H_2_O was added to the reaction solution and stirred for another 10 minutes. The reaction was started by addition of the initiator KPS (0.01 g dissolved in 1 mL DI H_2_O; 0.4 mM final concentration). The polymerization was allowed to proceed for 6 hours at 80°C under a nitrogen blanket. After reaction completion, the solution was cooled to room temperature using an ice bath and filtered through glass wool to remove traces of coagulum. Cross-linker-free microgels have been prepared additionally at 75, 85, and 90°C. Moreover, cross-linker-free microgels containing 0.01 mol-% methacryloxyethyl thiocarbomoyl rhodamine B have been prepared. These microgels are slightly larger than non-fluorescent microgels and have been used for AFM force mapping experiments. The final microgels were purified by dialysis (MWCO, 12–14,000 Da) against DI H_2_O for two weeks and were lyophilized before use. In this way unreacted monomers, initiator, and loosely attached polymer chains (sol fraction) were removed from the microgel suspension. Due to the increased polydispersity of microgels prepared in absence of cross-linker at a reaction temperature of 90°C (pOEGMA_90/0_), particles were subjected to three rounds of centrifugation before dialysis. The yield of particle formation for each particle type was determined gravimetrically after purification. Herein, the polymerization yields for the self-cross-linked particles were typically in the range of 80% and comparable to those prepared with PEG-DA.

### Microgel characterization

Dynamic light scattering (DLS, DynaPro, Wyatt Technology) was used to determine the hydrodynamic radii (*R*_H_) of the microgels dispersed in 10 mM HEPES, pH 7.4 and 25 mM HEPES/150 mM NaCl, pH 7.4. For DLS measurements, buffer solutions were filtered through a syringe filter prior experiment (Supor membrane, 0.2 μm pore width, PALL, Acrodisc). Light scattering data were collected at a scattering angle of 90° for 20 s per acquisition. A total of 25 acquisitions were collected and the measured intensity time correlation functions were analyzed using the cumulants method. From the resulting diffusion coefficients the hydrodynamic radii were calculated using the Stokes-Einstein equation.

Viscometry studies were performed in an Ubbelohde viscometer immersed in a temperature-controlled water bath. All measurements were done in 25 mM HEPES/150 mM NaCl, pH 7.4. The kinematic viscosity of dilute microgel suspensions was determined at 20°C by measuring the time it takes for the sample solutions of various microgel weight fractions for passing through the capillary. From the kinematic viscosity the zero shear viscosity of the colloidal suspension *η*_0_ and the solvent *η*_s_ can be derived. The ratio of both values, *η*_rel_, also known as relative viscosity, is related to the effective volume fraction *ϕ*_eff_ *via* an expression derived by Batchelor[44]

$$\eta_{rel}=\frac{\eta_{0}}{\eta_{s}}=1+2.5\phi_{eff}+5.9\phi_{eff}{}^{2}$$ (1)

Here, it is important to include the quadratic term into the equation in order to consider binary hydrodynamic interactions. The effective volume fraction in Eq 1 can be substituted with the term *kc* where *c* is the concentration of colloidal particles in wt/wt and *k* is a shift factor for converting the mass fraction to the effective volume fraction.

### Microgel deposition

For AFM imaging and AFM force spectroscopy microgels were deposited on 22 mm x 22 mm glass cover slips (VWR) according to a previously established procedure.[45] First, glass slides were cleaned by sonication in different solvents for 30 minutes for each case: dilute Alconox solution, acetone, ethanol, and isopropanol. The cleaned glass surface was silanized with 1% (v/v) APTMS in absolute ethanol on a table shaker for 2 h to render the surface cationic. The functionalized cover slips were stored in absolute ethanol until used. Before microgel deposition, the glass slides were rinsed with water, dried with nitrogen, and individually placed into wells of a six-well plate containing 25 mM HEPES/150 mM NaCl buffer solution, pH 7.4. The cover slips were equilibrated in the buffer solution for 30 minutes on a table shaker. After this period, the buffer solution was removed and replaced by a 0.25 mg/mL microgel solution. In case of 10 mol-% PEG-DA cross-linked microgels the particle concentration was varied between 0.001 and 0.25 mg/mL. Microgels were centrifugally deposited onto the glass surface at 20°C and 3700 rpm for 20 min using an Eppendorf centrifuge 5804 R equipped with a plate rotor. To form patterned microgel assemblies with 10 mol-% PEG-DA cross-linked microgels the centrifugation speed was varied between 500 and 3700 rpm. Afterwards the cover slips were gently rinsed with water to remove excess microgels and dried with N_2_.

### AFM imaging

Atom force microscopy (AFM) experiments were performed using a MFP-3D AFM (Asylum Research, Santa Barbara, CA). Images of microgels in air were collected in AC mode using conical shaped silicon AFM probes with Al reflex coating and a 42 N m^-1^ nominal force constant (Nanoworld, NCHR). In-liquid imaging was performed in 25 mM HEPES/150 mM NaCl buffer using silicon nitride probes with Cr/Au reflex coating and with nominal spring constants ranging between 0.03 and 0.26 N m^-1^ (Asylum Research, BL-TR400PB). Data were processed using the supplied software written in an IgorPro environment (Wavemetrics, Inc.).

### AFM force spectroscopy

AFM force mapping of microgels deposited on glass cover slips were performed in 25 mM HEPES/150 mM NaCl, pH 7.4 using high reflectance silicon nitride probes of conical shape with nominal spring constants ranging between 0.03 and 0.26 N m^-1^ and a tip radius of ~42 nm. Samples were allowed to equilibrate in 25 mM HEPES/150 mM NaCl, pH 7.4 buffer for approximately 30 min in the AFM chamber before analysis. The inverse optical laser sensitivity (InvOLS) and the spring constant (*k*_*s*_) of the AFM probe were calibrated by using the GetReal algorithm supplied with the MFP-3D software. This algorithm combines the Sader method with the equipartition theorem (thermal noise method) and allows the calibration of the InvOLS and *k*_*s*_ in one step without contact to the surface.[46] All force-distance measurements were performed in contact mode. Force maps with scan sizes of 2 μm x 2 μm were collected as a 32 x 32 array of force curves with trigger point set to 0.2 V. The force-distance curves were fitted with the Hertz model (Eq 2) *via* the MFP-3D analysis tools to extract the elastic modulus from the force mapping data. Because of the adhesive contact between the probe and the sample surface only the approach curves were analyzed.

### Tunable resistive pulse sensing

Nanopore translocation experiments were performed using a qNano (IZON Science, Oxford, UK) with thermoplastic polyurethane nanopore membranes (NP200) supplied by IZON Science Ltd. (NZ). According to the supplier, nanopores NP200 are suitable for solid particles of sizes ranging between 100 and 400 nm. Membranes were mounted on the jaws of the qNano and stretched to the optimum pore size for each measurement. Electrolyte solution (25 mM HEPES/150 mM NaCl, pH 7.4) was placed in both fluid cells, below and above the membrane, in order to establish a stable baseline current. For all qNano measurements, the electrolyte solution in the upper fluid cell was replaced by 40 μL of particles suspended in the same buffer solution. All measurements were conducted at 46.51 mm of applied stretch and a voltage of 0.34 V. Pressure was applied to the top fluid cell using IZON Science air-based variable pressure module (VPM). Current pulse signals were collected using the IZON propriety software. Particle sizes and number densities were determined by using a carboxylated polystyrene standard with a mean diameter of 212 nm and known number density as reference.

## Results and discussion

### Preparation of cross-linker-free p(OEGMA_300_-*co*-MAA) microgels and particle spreading at solid surfaces

OEGMA-based microgels have been polymerized under precipitation polymerization conditions, i.e. above the volume phase transition temperature (VPTT) of the forming particles, with varying cross-linker content ranging from 0 to 10 mol-% of PEG-DA. A small negative charge was introduced into the polymer network by incorporation of the sulfate groups of the initiator KPS and by addition of 2 mol-% MAA during polymerization. MAA was incorporated to easily deposit the particles on the positively charged surface for AFM studies. After completion of the synthesis the particles were cooled below the VPTT. The solution containing no PEG-DA was still turbid below the VPTT similar to the batches containing PEG-DA. This indicates that stable colloidal particles were formed from OEGMA_300_ and MAA without PEG-DA and that chain transfer reactions play a significant role in precipitation polymerization of oligo ethylene glycol methacrylates. To analyze self-crosslinking of microgels in more detail, and in particular the influence of polymerization temperature, we prepared microgels under cross-linker free conditions at varying reaction temperatures. Table 1 summarizes the details of the reaction conditions as well as physical properties of all microgels that have been synthesized for this study. All microgel particles were characterized by means of DLS to analyze their hydrodynamic radii *R*_H_. It is interesting to note that self-cross-linked microgels prepared at 85°C or lower temperatures but otherwise identical conditions are slightly smaller than microgels containing PEG-DA. This difference is attributed to cross-links formed by PEG-DA since it is well known that the choice and concentration of cross-linker have a large impact on the network properties.[47]

**Table 1:** DLS and viscometry data. Summary of results from DLS and viscometry studies for cross-linker-free and microgels cross-linked with varying amounts of PEG-DA. The symbol *R*_*H*_ and *k* represent the hydrodynamic radius of the particles and the shift factor in the Batchelor equation (Eq 1), respectively.

Sample^*a*^	Reaction temperature [°C]	Cross-linker [mol-%]	*R*_H_ in 25 mM HEPES/150 mM NaCl [nm]		*R*_H_ in 10 mM HEPES at 20°C [nm]	*k* at 20°C
			20°C	50°C		
pOEGMA_75/0_	75	0	239 ±8	205 ±5	n.a.	7.4 ±0.5
pOEGMA_80/0_	80	0	246 ±9	212 ±5	248 ±9	5.8 ±0.1
pOEGMA_85/0_	85	0	238 ±11	209 ±10	251 ±10	5.5 ±0.1
pOEGMA_90/0_	90	0	286 ±9	248 ±4	n.a.	n.a.
pOEGMA_80/1_	80	1	322 ±13	273 ±18	342 ±22	n.a.
pOEGMA_80/5_	80	5	300 ±±10	273 ±8	318 ±13	6.5 ±0.1
pOEGMA_80/10_	80	10	294 ±13	n.a.	291 ±14	6.0 ±0.1

^*a*^ The index of the sample names indicates the reaction temperature and amount of cross-linker.

**Fig 1:**
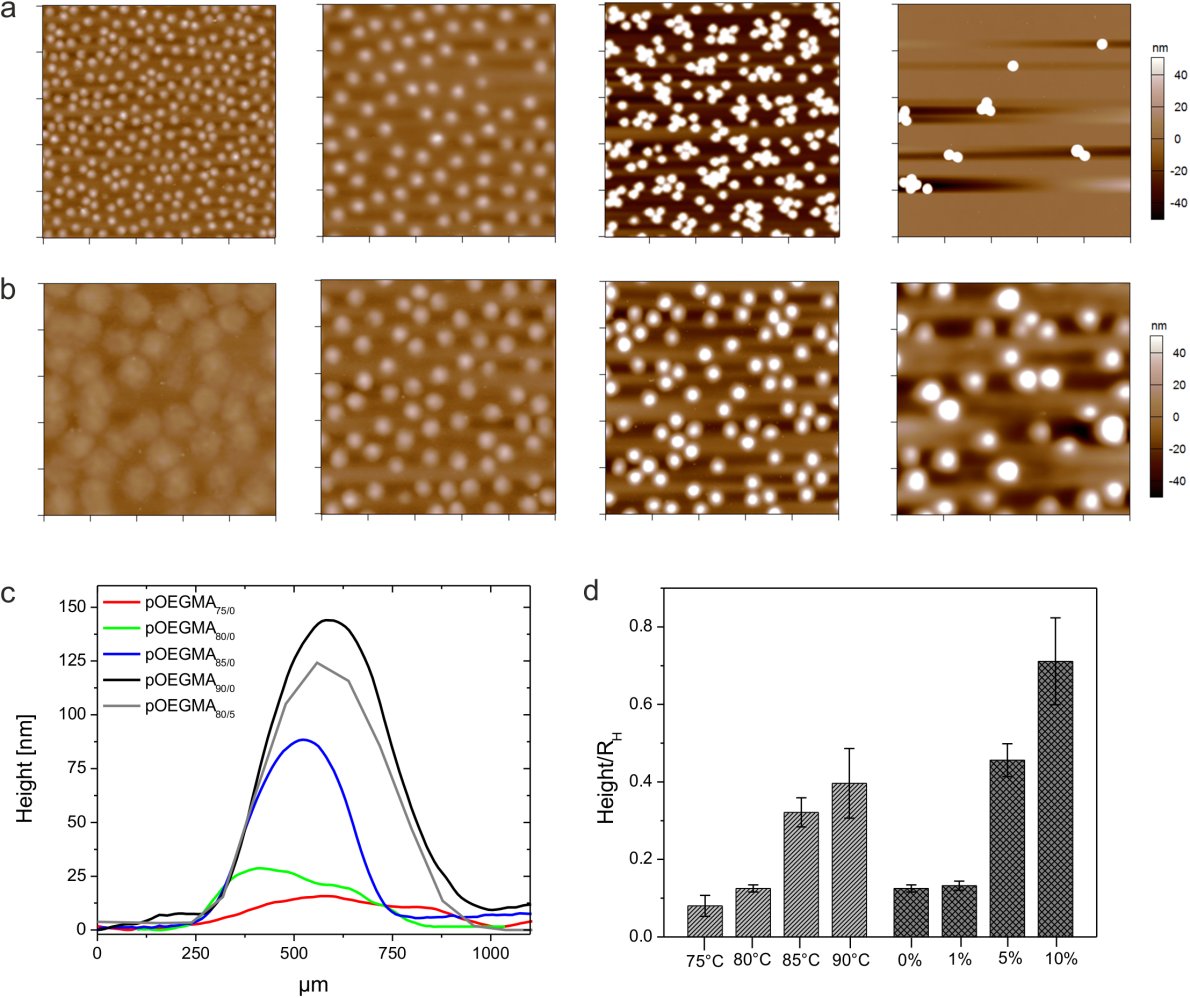
Microgel deposition and particle height analysis. AFM height retraces of deposited microgels after drying. Microgels were synthesized at 80°C and contain different amounts of PEG-DA. From left to right: 0, 1, 5, and 10 mol-% PEG-DA. Each image has a scan size of 10 μm x 10 μm. (a). AFM height retraces of cross-linker-free microgels prepared at different polymerization temperatures. From left to right: 75, 80, 85, and 90°C. Each image has a scan size of 5 μm x 5 μm (b). The corresponding height profiles of the particles show the influence of cross-linking density on particle spreading (c). Panel (d) shows the particles heights after normalization to the corresponding hydrodynamic radii *R*_H_. Errors were calculated *via* error propagation from the standard deviations derived for the AFM particle height and *R*_H_.

In order to characterize particle softness and deformability we investigated particle spreading on cationic amine-functionalized glass surfaces. Fig 1A displays AFM height retraces of dried particles that vary in their PEG-DA content from 0 to 10 mol-%. According to these images and the particle height/hydrodynamic radius ratios in Fig 1D, microgel particles with 0 mol-% PEG-DA appear to spread on the surface to higher extent than microgels cross-linked additionally with 5 mol-% PEG-DA or higher, indicating a higher network flexibility of cross-linker-free particles. Moreover, AFM images of self-cross-linked microgels prepared at different temperatures (Fig 1B) demonstrate that the temperature is a useful parameter to change the mechanical properties of cross-linker-free hydrogels. Cross-section analysis of these particles shows that particle height profiles strongly depend on the reaction conditions during polymerization (Fig 1C). The average height of particles prepared at 75°C is as low as 19 nm whereas particles prepared at 85°C show particle heights of ~77 nm. For comparison, 5 mol-% PEG-DA cross-linked microgels are characterized by a height of ~137 nm. In Fig 1D the heights of all particles have been normalized to the hydrodynamic radius to take slight variations of particle sizes into account.

**Fig 2:**
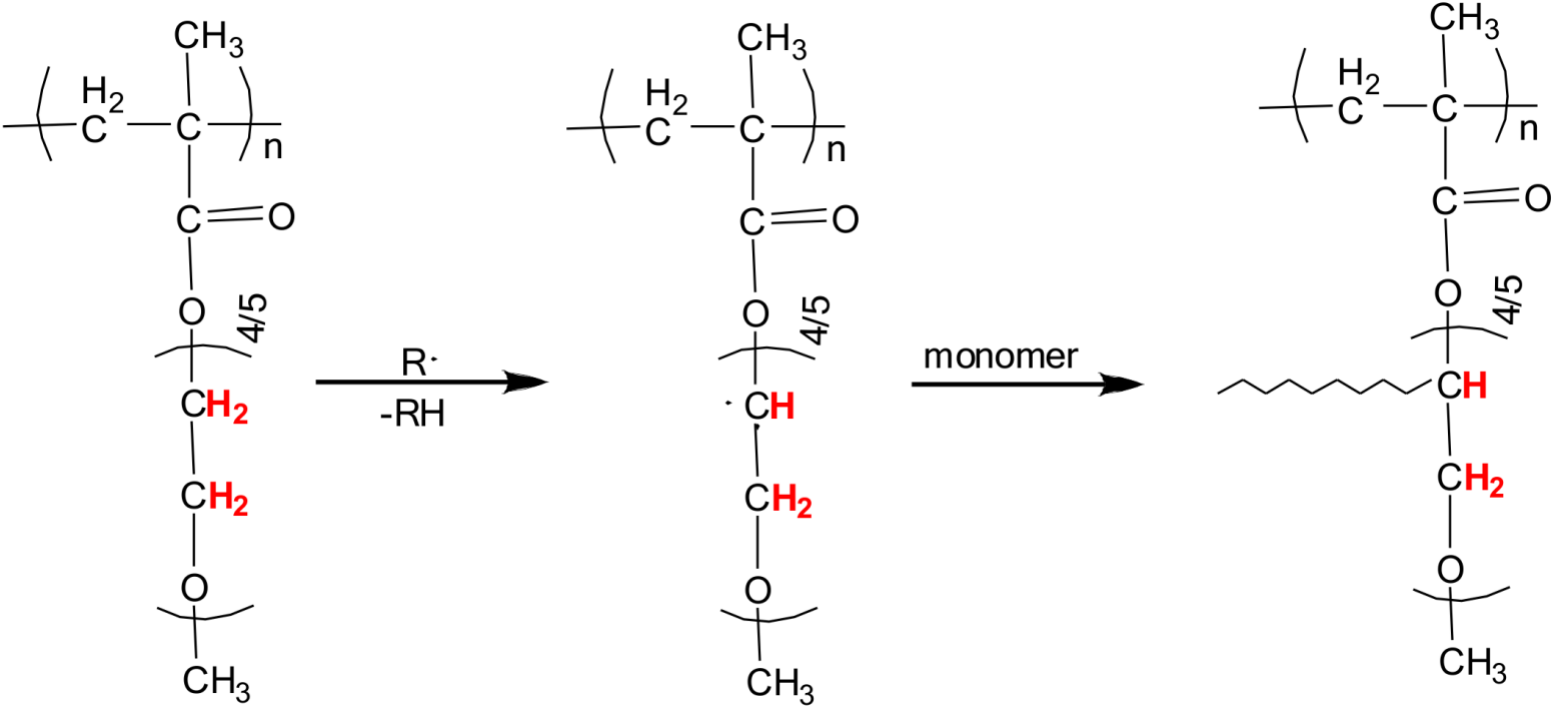
Chain transfer reactions. Schematic representation of cross-linking *via* chain transfer reactions originating from the oligo ethylene glycols sidechains in pOEGMA microgels. Hydrogen atoms labeled in red can be abstracted by the attack of a radical that leads to the formation of midchain radicals. These radicals can attack other monomers or growing polymer chains, which results in branching and cross-linking.

Branching and self-crosslinking during free radical polymerization is a well-known process for acrylic and vinylic monomers, such as *N*-vinylformamide, alkyl acrylates and NiPAm, and occurs *via* formation of mid-chain radicals on the polymer backbone.[20–22, 26, 48] These species are generally formed through hydrogen atom abstraction events yielding tert-C free radicals that are stabilized by their conjugation to adjacent groups. In addition to hydrogen transfer originating from the polymer backbone, sometimes side-chain transfer products need to be considered as well.[26] In case of methacrylates, chain transfer reactions from the polymer backbone are highly unlikely due to the lack of active hydrogen atoms that can be abstracted by other radicals.[22] For example, Wang et al. investigated the polymerization kinetics of various monomers, including acrylate, styrene, and methacrylate. At polymerization temperatures lower than 80°C, between 80 and 180°C, and above 180°C backbiting, i.e. mid-chain radical formation, could be observed for the polymerization of acrylate but not for methacrylate.[49] This is strong evidence for the absence of abstractable tertiary hydrogens in the main chain of polymethacrylates. On the other hand ether groups contain easily abstractable hydrogens as this was shown in the radical polymerization of methacrylate monomers containing alkylether functionalities under air.[50] Thus, the formation of stable microgel particles under cross-linker free conditions is most likely established through crosslinking events initiated by chain transfer reactions from the oligo ethylene glycol sidechains of OEGMA. As illustrated in Fig 2, H abstraction at the secondary C atoms results in mid-chain radicals that are stabilized through the O atoms in proximity. These radicals are able to attack monomers or other growing polymer chains leading to branching and cross-linking. Due to the presence of several ethylene glycol units per monomer, repeated H abstraction and branching might take place resulting in the formation of a stable microgel network.

Moreover, chain transfer reactions take place more frequently as the temperature is raised.[49] Thus, microgels prepared at higher temperatures are characterized by higher cross-linking densities and presumably by less flexible polymer networks. Interestingly, self-cross-linked particles prepared at 90°C show comparable spreading to those particles prepared at 80°C with 5 mol-% PEG-DA indicating comparable cross-linking contents (Fig 1D). These observations demonstrate that cross-linking *via* chain transfer reactions is very efficient in the case of OEGMA-based microgels, as opposed to its relative inefficiency in the case of pNiPAm.[21] This finding is, however, not very surprising when considering the number of reactive hydrogen atoms per oligoethylene glycol side chain that can be attacked by free radicals. In case of OEGMA_300_, eight to ten sites per side chain are available to initiate self-cross-linking. For statistical reasons, the cross-linker density of PEG-DA-free OEGMA-based microgels is likely higher than the network density of self-cross-linked pNiPAm microgels prepared at comparable reaction conditions. Thus, the particles prepared here do not exhibit the same degree of deformability as pNiPAm microgels. However, cross-linker-free OEGMA-microgels particles prepared at low reaction temperatures are still highly deformable as shown in their enhanced spreading diameters after surface deposition (Fig 1B and 1C). At this point, it becomes obvious that cross-links formed through chain transfer reactions contribute strongly to the overall network density. As can be seen from the particle height/hydrodynamic radius ratios in Fig 1D, chain transfer processes at the polymer chains dominate the cross-linking process at low concentrations of crosslinking agent and neglecting this mechanism would lead to significant underestimation of the net cross-linking density and to misinterpretation of the microgel network structure.

### Hydrolysis of cross-links in self-cross-linked microgels

To further investigate our hypothesis that network formation in absence of cross-linker occurs *via* chain transfer reactions originating from the oligo ethylene glycol side-chains, we analyzed the time-dependent degradation of self-cross-linked microgels OEGMA_80/0_ in acidic medium at 50°C. Under these conditions the ester bonds that covalently bond the sidechains to the polymer backbone are prone to acid catalyzed hydrolysis. In the case that cross-linking occurs solely *via* the oligo ethylene side-chains, self-cross-linked polymer networks should be degradable under these conditions. To test this hypothesis, small aliquots were withdrawn at certain time points and diluted in 25 mM HEPES/150 mM NaCl buffer to deposit partially degraded microgels and polymer fragments on amine functionalized glass slides. Due to the small negative charge introduced into the microgel during synthesis, the particles adsorb to the surface with sufficient affinity for analysis. The surfaces were imaged in air using AFM. AFM imaging of deposited microgels[51] has been demonstrated to be a useful method to qualitatively analyze degradation process and the material loss as polymer chains are cleaved from the microgel network. It should be noted that the particle radius determined by light scattering did not decrease with the onset of degradation but showed a small increase due to network swelling as consequence of the cleavage of cross-links. Similar obeservations have been found for other degradable microgel systems [52]. From this, we decided to analyze the degradation process by AFM of single deposited particles to obtain more direct visualization of particle degradation.

**Fig 3:**
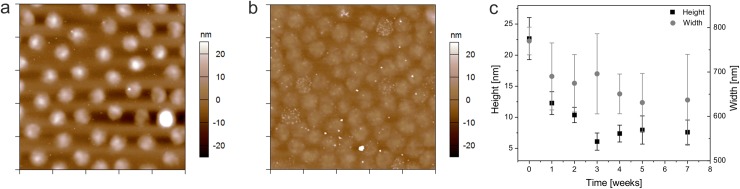
Microgel degradation. AFM height retraces of cross-linker-free microgels pOEGMA_80/0_ before (a) and after (b) incubation in 3 M HCl at 50°C for 7 weeks. Each image has a scan size of 5 μm x 5 μm. Particle heights and spreading diameters of partially hydrolyzed microgels deposited on glass substrates are plotted as function of incubation time (c).

Fig 3A and 3B show AFM height retraces of a monolayer of OEGMA_80/0_ before incubation in the acidic environment and after an incubation period of 7 weeks, respectively. The particle heights and spreading diameters were analyzed and are plotted as function of incubation time in Fig 3C. Microgels degrade significantly over a period of two months when incubated in acidic environment as demonstrated by the reduced particle heights: microgel height decreases by 67% over this period. Moreover, the particle roughness increases as the particles degrade (S1 Fig). A close look at the AFM image of progressively degraded particles (Fig 3B) reveals that many microgels contain nano-sized particulate matter, which leads to a spotted appearance of the microgels in the AFM height retrace. Cleaved polymer fragments that are trapped inside the remaining microgel network are the most likely origin of this observation. Retarded diffusion of debris outside the polymer network might slow down the degradation process and delay complete hydrolysis to longer time scales. However, these data strongly suggest that chain transfer reactions dominantly take place at the oligo ethylene glycol sidechains and not at the polymer backbone.

### Swelling behavior of microgels in solution

**Fig 4:**
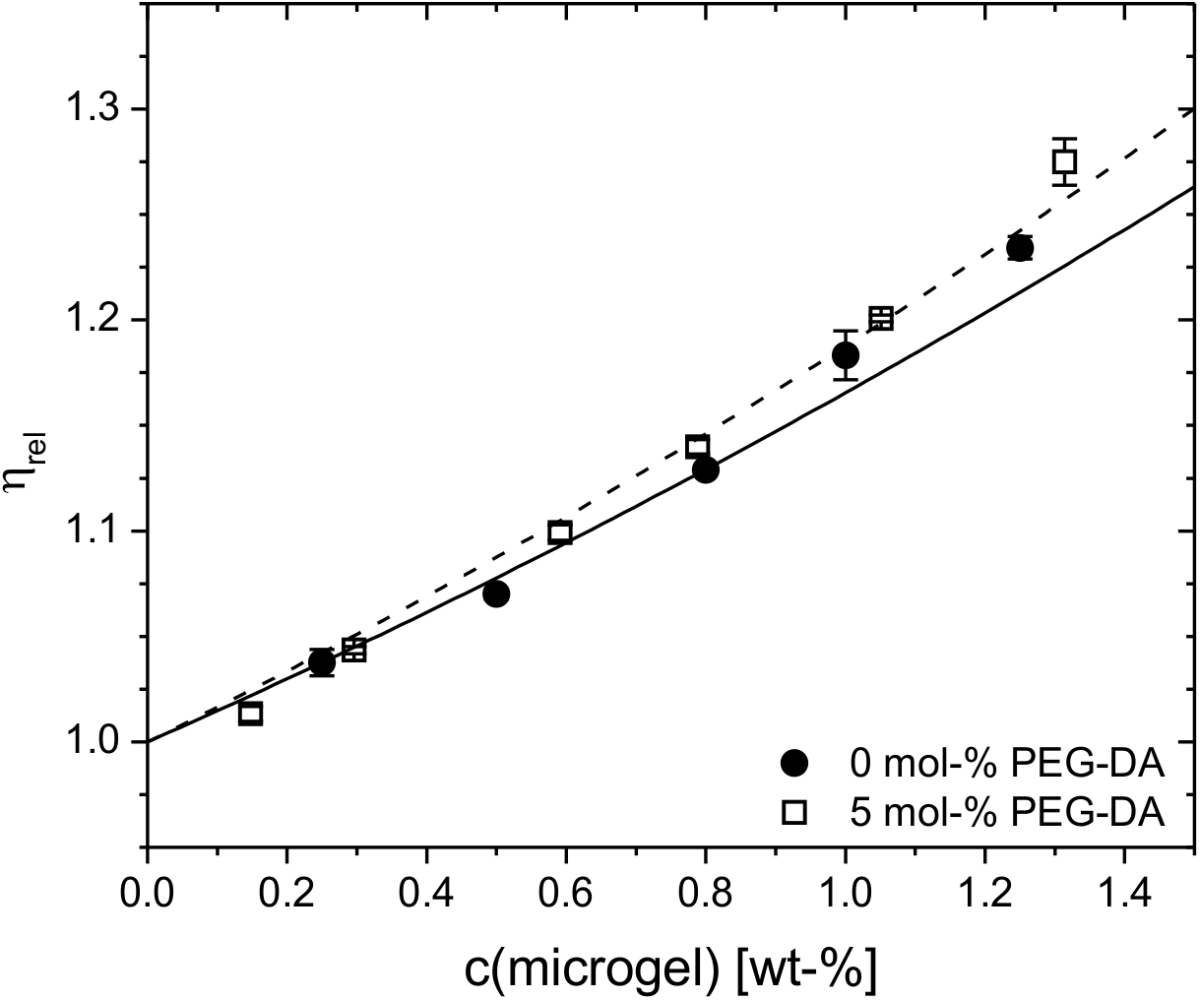
Viscosity data. Concentration dependence of the relative viscosities *η*_rel_ of cross-linker-free (pOEGMA_80/0_) and 5 mol-% PEG-DA (OEGMA_80/5_) containing microgels prepared at 80°C. The solid and dashed lines represent fits according to Eq 1.

As shown previously in case of pNiPAm microgels, different degrees of cross-linking should result in different degrees of swelling that entail marked differences in the inner segmental densities.[47] To determine the polymer concentration inside the particles *c*_p_, we need to determine the effective volume fraction *ϕ*_eff_ of the microgel particle dispersions. A simple method to derive *ϕ*_eff_ is to employ the relative viscosity *η*_rel_ of the colloidal suspension in the dilute regime. The relative viscosities of dilute microgel solutions of pOEGMA_80/0_ and pOEGMA_80/5_ are shown in Fig 4 as function of the mass concentration. The experimental data was fit by using the Batchelor equation (Eq 1) to extract the shift factor *k*. Using the values of *k* together with the knowledge of the particle volume yields the polymer content of the particle *c*_p_.

The *k*-values of all microgels are found to be in the range of 5.5 and 7.5 which converts to a polymer concentration of *c*_p_ ~ 160 g/L. Obviously, the effective volume fraction of OEGMA-based microgels does not show significant dependence on the cross-linker content. These findings, *i*.*e*., the high polymer content and the invariance of the volume fraction, are different from observations made in case of pNiPAm microgels. For pNiPAm microgels a strong decrease of the *k*-value, and thus, increase of *c*_p_, has been found when the amount of cross-linker was changed from 0.5 to 5 mol-%.[47] Moreover, the *k*-values of OEGMA-based microgels are significantly smaller than those derived for pNiPAm microgels indicating smaller effective volume fractions and denser polymer networks of OEGMA-based microgels. According to these observations we can conclude that the swelling of OEGMA microgels is not solely restricted by the elasticity of the network resulting from the cross-linking points of the network but rather is significantly controlled by the mixing contribution of the osmotic pressure, *i*.*e*., by the interplay of polymer-solvent and polymer-polymer interactions.

### Elastic moduli of adsorbed microgel particles

As cross-linker-free microgels show enhanced spreading compared to PEG-DA cross-linked particles, we expect significant differences of the elastic moduli of the microgels. For this analysis we selected two microgels, OEGMA_80/0_ and OEGMA_80/5_, to investigate differences of the mechanical properties with spatial resolution at the single particle scale. Nanoindentation data were gathered by high-resolution force mapping on single deposited microgel particles. The measurements were done after rehydration in 25 mM HEPES/150 mM NaCl buffer, pH 7.4, *i*.*e*., in the hydrated state of the microgels. The data is represented by a log-log plot of the loading force imposed by the AFM tip as function of the measured indentation (S2A and S2B Fig). We measured force-indentation curves up to 1 nN of applied force. This rather small force threshold still provides enough signal well above the noise while avoiding significant influence of the underlying solid support. Assuming a conical AFM tip geometry, the Hertz-Sneddon equation describes the relation between the loading force *F* and the indentation *δ* as follows

$$F=\frac{E_{Y}}{1-\nu^{2}}\frac{2\tan\alpha }{\pi }\delta^{2}$$ (2)

where *E*_Y_ is the Young´s modulus, *ν* is the Poisson ratio, and *α* is the semi-opening angle of the conical indenter. Studies done by Voudouris et. al demonstrate that microgels below and above the VPTT behave very closely to incompressible isotropic elastic material.[53] For this reason the Poisson ratio was set to 0.5 for the experiments done this study. According to Eq 2, the Hertz model predicts a constant elastic modulus throughout the measurement. Indeed, this behavior has been found for force spectroscopy studies of single microgel particles on solid supports for small indentations and low forces.[11, 54, 55] The log-log plots of the force-indentation curves monitored for the self-cross-linked and PEG-DA cross-linked particles, both collected at the particle center and the periphery of the microgel, show a linear relationship over large regions of the plotted data (S2A and S2B Fig). For small indentations, linear fits give slopes in the order of 2, which confirm that the experimental data is sufficiently described with the Hertz model (Eq 2). Fitting the force-deformation curves at the particle apex using Eq 2 leads to Young´s moduli of (430 ±51) kPa for pOEGMA_80/5_ and (80 ±35) kPa for cross-linker-free particles pOEGMA_80/0_. According to these results, E_Y_ at the particle center of cross-linker-free microgels is more than 5 times smaller than that of microgels containing 5 mol-% PEG-DA. This observation demonstrates the higher deformability of cross-linker-free particles and explains their ability of enhanced spreading.

**Fig 5:**
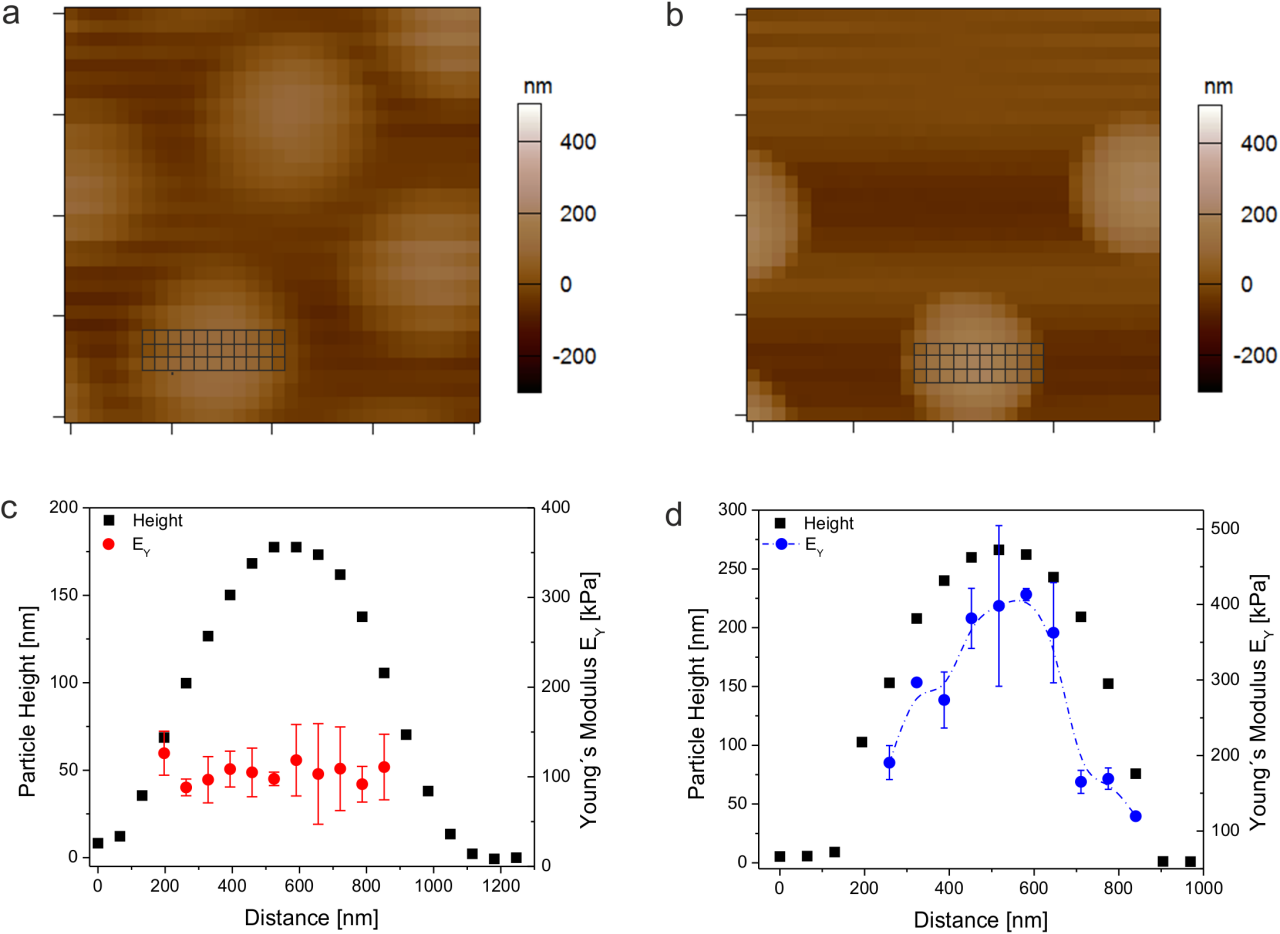
AFM force mapping. AFM topography maps of cross-linker-free microgel particles pOEGMA_80/0_ (a) and 5 mol-% PEG-DA cross-linked particles pOEGMA_80/5_ (b) after rehydration in 25 mM HEPES/150mM NaCl, pH 7.4. Each image has a scan size of 2 μm x 2 μm. For every square a complete force-distance curve was collected. The grids placed on top of the particles indicate the squares used for analysis. Particle heights and the corresponding Young´s moduli are plotted versus the particle cross section of microgels pOEGMA_80/0_ (c) and pOEGMA_80/5_ (d).

In order to spatially resolve the mechanical properties, we need to extend the analysis of Young´s moduli over the entire microgel surface. Therefore, we evaluated a complete set of force-indentation curves across the microgel profile. Force mapping measurements were performed with grid spacing (63 nm) larger than the tip radius (~42 nm) to adequately resolve the sample stiffness as function of the tip position. As indicated in the height maps of both particles in Fig 5A and 5B, three sets of force-deformation curves across each particle were evaluated to calculate the spatial-resolved elastic moduli. The corresponding values of the Young´s moduli are plotted together with the topography maps of the particles (Fig 5C and 5D). In case of PEG-DA cross-linked particles we find decreasing elastic moduli towards the periphery of the microgel. This analysis was repeated for several individual microgel particles (S3 Fig) demonstrating a decrease of the Young´s modulus from the particle center towards the shell by a factor of ~2.2. These findings are comparable to lateral stiffness gradients observed in case of pNiPAm microgels.[11, 18] The variation of the elastic properties across the microgel surface is likely caused by structural inhomogeneities resulting from mismatched reactivity ratios between cross-linker and monomer. Due to the faster polymerization rate of PEG-DA compared to OEGMA,[56] microgels cross-linked by PEG-DA most likely consist of a higher cross-linked core and loosely cross-linked shell as well leading to stiffer regions at the particle center and softer regions at the periphery. In contrast, no large variations of the elastic moduli were found in case of pOEGMA_80/0_ (Fig 5C and S3 Fig). The elastic moduli measured at various points of the self-cross-linked network scatter around the same value of 80 kPa. This trend was found for repeated measurements with different particles (S3 Fig). These findings demonstrate that particle formation in absence of a PEG-DA results in more homogenous network structures. This finding is supported by confocal microscope images from micron-sized and fluorescently labeled ultra-low cross-linked (ULC) pNiPAm microgels prepared under similar conditions than the OEGMA particles. These images show that the particles are homogenously labeled by fluorescent dyes indicating a homogenous network structure.[10] Recently, Richtering *et al*. investigated the structure of ULC pNiPAm microgels by static light scattering.[57] Therein, the particles were prepared *via* non-stirred precipitation polymerization using KPS as initiator. The scattering experiments of these particles suggest an inverted cross-linking structure having the highest polymer density close to the particle surface. The authors explain this finding by hydrogen abstraction reactions and subsequent cross-linking at the particle surface by persulfate initiator radicals during the late stages of the polymerization and hindered diffusion of radicals to the core of the growing particles. However, the experiments performed in our study do not give any indication for a core-depleted particle structure. It is known that the polymerization conditions have a significant influence on the polymerization process as well as on particle growth. In ref. [57] particles were prepared at much higher monomer and initiator concentrations than in this study. Moreover, particles herein were prepared by precipitation polymerization under constant stirring, whereas the batches in ref. [57] were prepared *via* non-stirred precipitation polymerization. From the results shown here we conclude that chain transfer reactions *via* hydrogen abstraction by radicals take place throughout the polymerization process and proceeds during particle growth starting at the early stage of particle formation.

### Translocation of microgels through nanopores

**Fig 6:**
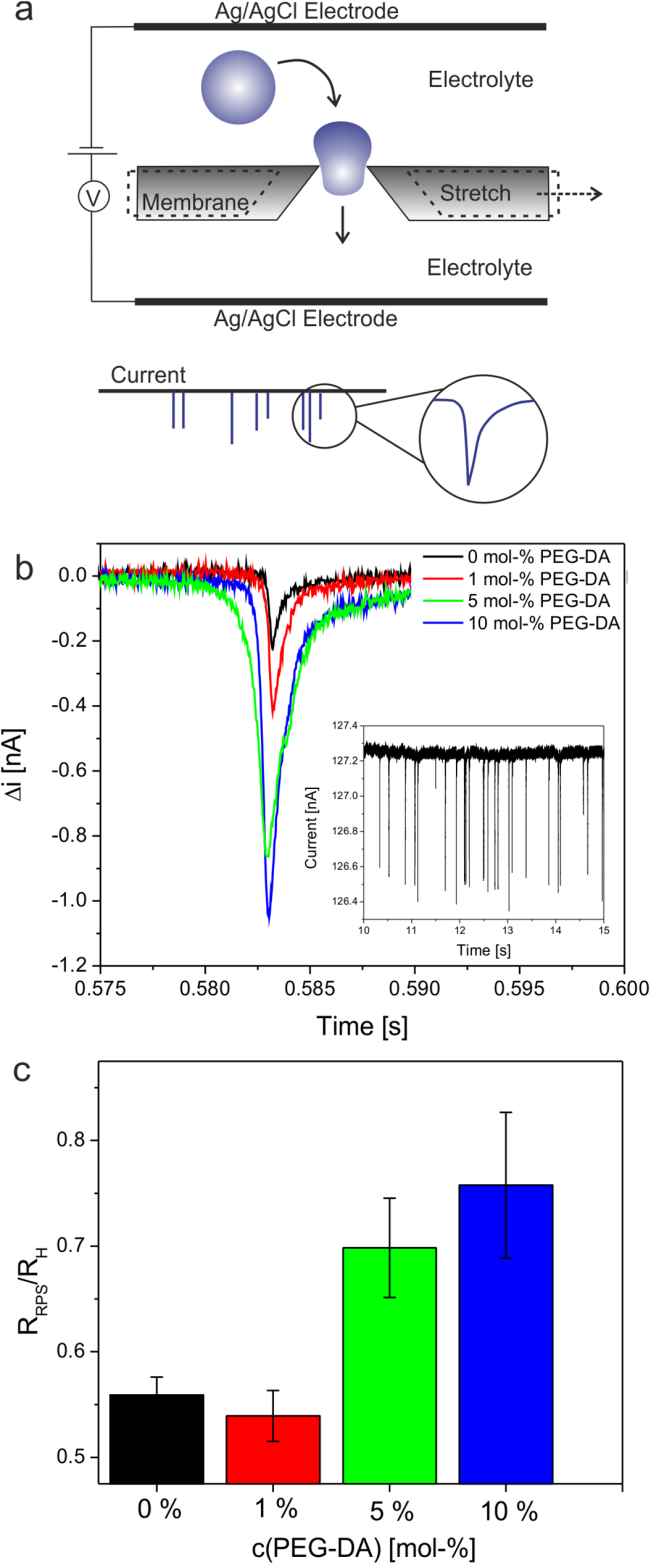
Nanopore translocation of microgels. Schematic description of the RPS technique using resizable elastomeric nanopores (a). Overlay of the current pulse signals generated by cross-linker-free particles and microgels cross-linked by 1, 5, and 10 mol-% PEG-DA (b). The inset shows the recording of multiple pulse signals generated by 5 mol-% cross-linked particles. Ratio between the particle sizes determined by RPS and DLS (c).

To further characterize the microgel particles regarding their size, flexibility, and network density we used resistive pulse sensing (RPS).[58–61] This single-particle sensing technique is based on the Coulter principle; it involves the detection of temporal changes of the current flow across a pore sensor where the changes are associated with the pressure-driven convective and/or electrokinetic driven translocation of particles through the pore.[59, 62] Particles dispersed in a solution of high ionic strength traversing the pore are detected as a decrease in the ionic current, which is denoted as a blockade event with its amplitude being proportional to the excluded volume of the particle (Fig 6A). Thus, the blockade events contain information on particle size (pulse magnitude) and velocity (pulse duration).

In this study, we use resizable elastomeric nanopores of conical shape. Fig 6B displays the blockade events induced by the passage of cross-linker-free microgels and microgels containing 1, 5, and 10 mol-% PEG-DA as crosslinking agent, through a nanopore with an orifice that is smaller than the hydrodynamic diameter of the particles. The experiments described above indicate that self-cross-linked particles show high polymer network mobility, which results in lower elastic moduli and extensive spreading on surfaces. Thus, we speculated that due to their low network density 0 mol-% PEG-DA cross-linked microgels are highly deformable and would be able to translocate through nanopores that are smaller than their hydrodynamic radius. Indeed, self-cross-linked microgels and 1 mol-% PEG-DA cross-linked particles generate significantly smaller and shorter blockade pulses than microgels of similar hydrodynamic diameter but higher cross-linking densities. For self-cross-linked and 1 mol-% PEG-DA cross-linked microgels the physical radius calculated from the current pulses is significantly smaller (~50%) than the hydrodynamic radius (Fig 6C). Also 5 and 10 mol-% cross-linked microgels have physical radii slightly smaller (20–30%) than their hydrodynamic diameter. A reasonable explanation for this observation is twofold. First, DLS provides information about the overall size including dangling polymer chains in the outer shell of the microgels whereas these dangling polymer chains do not contribute to the signal in RPS. Secondly, it is known that RPS provides accurate values for the physical diameter of solid particles while it underestimates the size of porous particles.[63] The reason for this is that the pores within the particle are filled with electrolyte solution in which the particles are suspended for the measurement. This increases the particle conductivity and, thus, decreases the magnitude of the resistive pulse from which the particle volume is derived. Microgels are swollen by the solvent and contain a large amount of the electrolyte in which they are suspended. Similar to porous particles, the drop of current during particle translocation is lower than for solid particles because of the increased conductivity caused by the electrolyte within the microgel, which results in smaller observed physical radii.

From the viscometry studies that indicate comparable volume fractions (polymer mass fractions) of all microgels and the translocation data we conclude that the pronounced decrease of pulse magnitudes of cross-linker-free and 1 mol-% PEG-DA cross-linked microgels is likely due to significant deformation of the polymer network upon pore entry. It is also important to note that increasing the applied pressure reduced the time it took particles to traverse the pore as shown by the decreased pulse durations (S4A Fig). However, variable pressures did not alter the pulse magnitudes suggesting that a change of applied pressure does not affect the deformation of particles as they pass through the pore (S4B Fig). Additionally, the decreased pulse signals found for cross-linker free microgels compared to highly cross-linked particles may also indicate higher electrolyte mobility in gels of low network density and, thus, a higher contribution of the ions in these gels to the overall conductivity.

### Surface patterning

**Fig 7:**
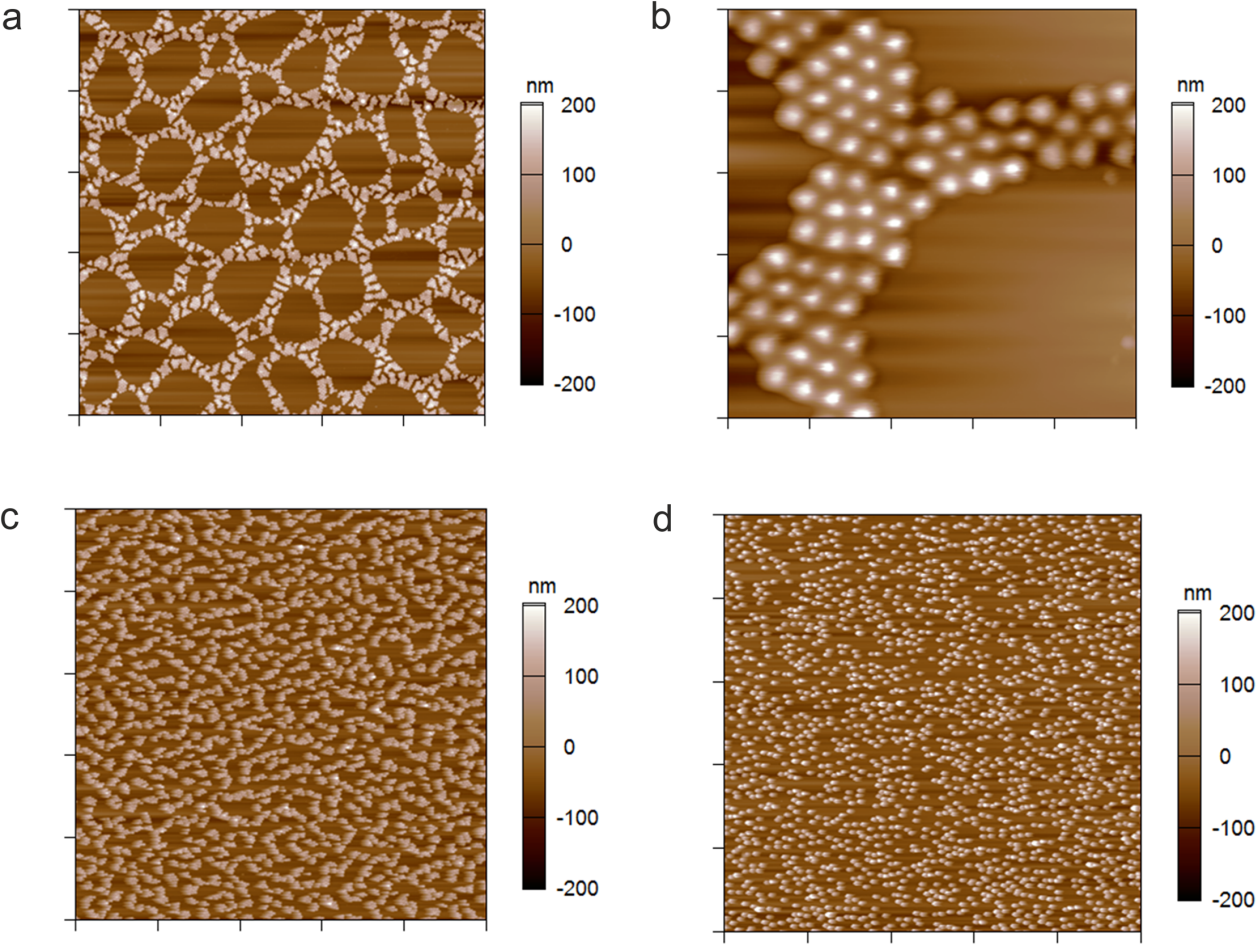
Formation of patterned particle assemblies. AFM height retraces in air of 10 mol-% cross-linked microgels deposited on APTMS-functionalized glass from a solution of 0.25 mg/mL pOEGMA_80/10_ in 25 mM HEPES/150 mM NaCl (a, b), 10 mM HEPES (c), and DMSO (d). Images in panels (a), (c), and (d) have a scan size of 50 μm x 50 μm and the image in panel (b) has a scan size of 10 μm x 10 μm.

So far, we have demonstrated that the cross-linking density is important for controlling physical properties of single particles, such as spreading and nanopore translocation. Additionally, we have found that particle deformability is a key parameter for tuning the assembly of particles on solid substrates into self-organized patterns. As shown in Fig 7A, centrifugal deposition of particles containing 10 mol-% PEG-DA from a solution of 25 mM HEPES/150 mM NaCl results in the formation of cellular networks resembling those obtained in dewetting polymer systems[64]. In contrast, highly deformable particles have a high affinity to spread on the substrate forming dense particle monolayers that do not show any ordering effects. The networks formed from stiff particles are characterized by a well-defined correlation length and a narrow distribution of large cells with a minor fraction of smaller cells.

By analogy to dewetting phenomena observed for polymer thin films,[64] patterning of colloidal suspensions is caused by a complex interplay of different hydrodynamic and interfacial forces. We attribute the exceptional ordering effect observed in case of stiffer particles to a delicate interplay of inter-particle and particle-surface interactions as well as frictional forces, interfacial tension between the substrate surface and the liquid and particle mobility (resulting from particle-surface interactions). Besides particle composition, the deformability of the particle is a convenient tool to modify these interactions. Increasing the particle stiffness increases the interfacial tension and reduces the contact area between the particle and the substrate. The limited particle spreading reduces the attractive electrostatic attractive interaction between the negatively charged microgel and the positively charged APTMS-functionalized glass surface due to a reduced number of acrylic acid groups that are able to be in contact with the oppositely charged substrate.

This weakens the electrostatic interaction towards the substrate and increases the particle mobility enabling the formation of cellular particle networks. In addition, long-range interactions, such as repulsive electrostatic forces between the microgel particles induce particle ordering and the formation of crystalline domains (Fig 7B). The magnitude and length scale of the attractive and repulsive electrostatic interactions can be further modified by the ionic strength of the surrounding system and the choice of solvent, *i*.*e*., the solvent dielectric constant. For example, particle deposition from buffer of low salt concentration leads to worm-like structures (Fig 7C). Increasing the strength of electrostatic interactions even further by changing to a solvent with smaller dielectric constant than water, *e*.*g*., DMSO, prevents particle dewetting completely; indeed, particles dispersed in DMSO form monolayer structures characterized by random particle deposition (Fig 7D). Furthermore, the final morphology of the self-assembled particle pattern can be tuned by the particle concentration and the centrifugation speed during the deposition process (S5A and S5B Fig). Decreasing particle concentrations as well as centrifugal forces result in less ordered particle patterns, such as worm-like structures, and at the smallest centrifugation speed and particle concentration to the random deposition of single particles.

Similar patterns to those shown here have been observed in case of gold nanoparticle films, which have been produced by spin-coating from an organic solvent.[65] Monte Carlo simulations of the formation of cellular patterns during solvent evaporation suggested that, although the cell positions were spatially correlated, the pattern formation results from a stochastic nucleation-driven dewetting process.[65] Since the particles used here as well as the deposition process differ significantly from the work presented in Ref [65], other formation mechanisms, like long-range ordering processes, including spinodal decomposition, might be plausible explanations for the formation of spatially correlated networks as well.

**Fig 8:**
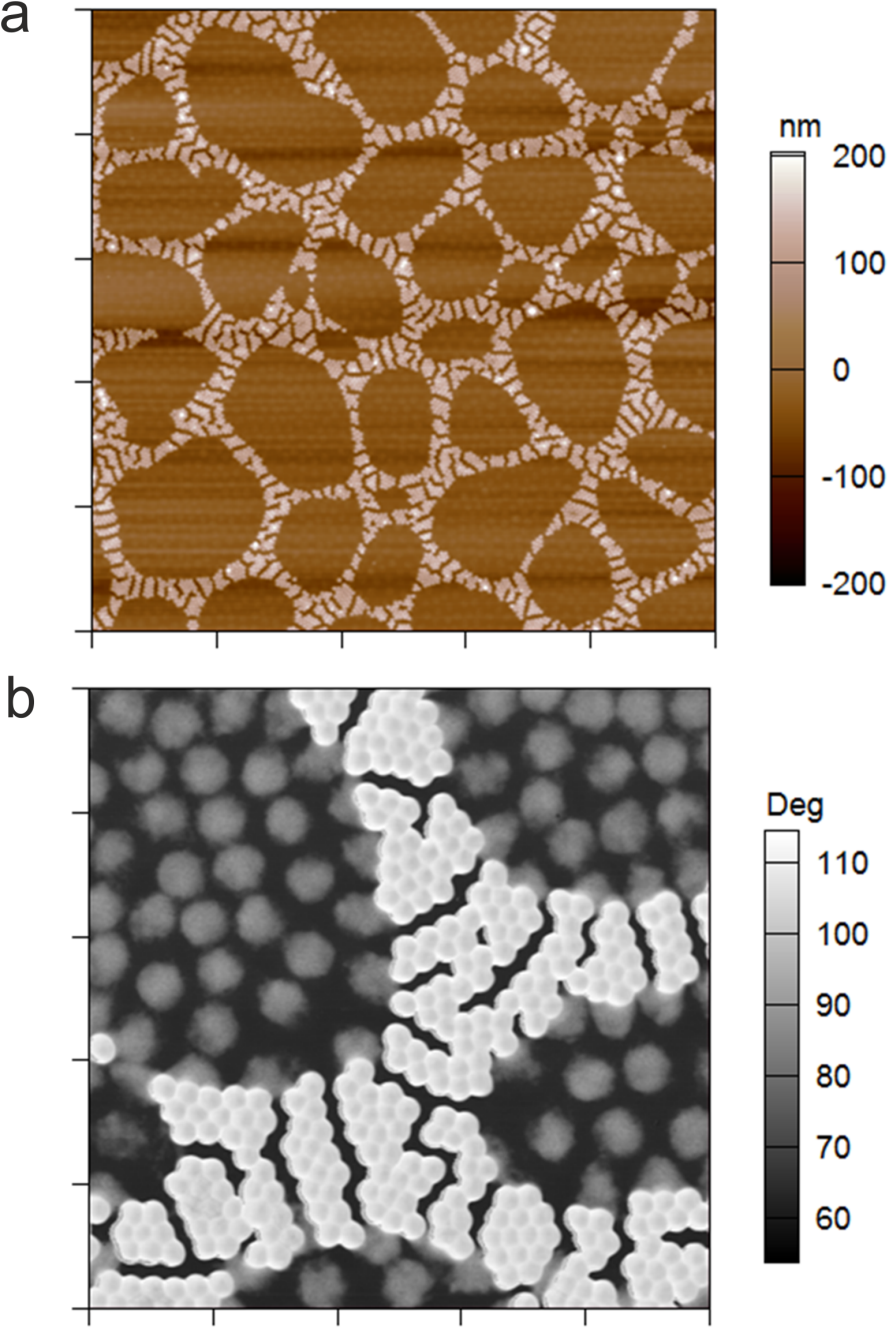
Patterned particle monolayer prepared from soft and stiff microgel particles. AFM height retrace of a patterned microgel monolayer formed upon sequential deposition of stiff microgels pOEGMA_80/10_ and soft microgels pOEGMA_75/0_ on an APTMS-functionalized glass substrate. Scan size: 50 μm x 50 μm (a). AFM phase retrace of the patterned microgel monolayer recorded at a scan size of 10 μm x 10 μm (b).

As shown in Fig 8 self-assembled patterns can be subjected to another rounds of particle deposition to fill the empty voids in the cellular networks with another particle type. Using highly deformable cross-linker-free microgels pOEGMA_75/0_ for the second deposition step, it is possible to create spatially resolved soft domains separated by a network of stiffer particles (Fig 8A and 8B). The AFM phase retrace displayed in Fig 8B highlights the patterned assembly of soft and stiff microgels. We believe that the controlled self-assembly of microgels with varying deformability offers a promising route for the bottom-up buildup of 2D-structures with patterned mechanical properties.

## Conclusion

In this study we have demonstrated that it is possible to prepare microgels based on poly/oligo(ethylene glycol) side-chain monomers without the use of bifunctional co-monomers as cross-linking agent. By analogy to pNiPAm microgels, the formation of stable particles is established through chain transfer reactions *via* the formation of mid-chain radicals. Network formation and deformability is controlled by the reaction temperature during the polymerization process. At low reaction temperatures chain transfer reactions are less likely to occur enabling the formation of loosely cross-linked hydrogel particles with high degrees of deformability. This is shown, for instance, in the enhanced spreading of particles deposited onto solid surfaces as detected by AFM. Additionally, AFM nanoindentation experiments demonstrate that PEG-DA-free microgels are characterized by elastic moduli that are significantly smaller than PEG-DA cross-linked microgels prepared at otherwise identical conditions; the elastic modulus at the particle center of self-cross-linked microgels is ~5 times smaller than the value found microgels containing 5 mol-% PEG-DA. Moreover, cross-linker-free microgels are found to have more homogenous network structures since spatially resolved studies indicate that the elastic modulus of these particles is constant across the whole particle cross-section. In contrast, particles containing PEG-DA show significant stiffness gradients between the center and the periphery of the particle.

In addition, we performed particle degradation studies to identify active sites that participate in chain transfer reactions. This analysis demonstrates, that cross-linking dominantly occurs at the acid cleavable oligo(ethylene glycol) sidechains. Hydrogen atoms at the secondary C-atoms of the ether groups can be abstracted by free radicals to form stabilized mid-chain radicals. These radicals are able to react with monomers or propagating polymer chains that inevitably results in the formation of cross-links. Since the outcome of cross-linking depends on the number of reactive groups in the monomer, the length of the side-chain, *i*.*e*., the number ethylene oxide units, is another important parameter to control the cross-linking density and network properties.

We have also shown that network deformability is a crucial parameter for the translocation of microgels through nanopores. Microgels with high levels of deformability traverse pores that are smaller than the particle diameter presumably by deforming their network as particles pass the nanopore. Moreover, network flexibility defines particle-particle as well as particle-surface interactions. This allows us to form controlled particle dewetting patterns and the build-up of two-dimensional self-assembled structures from particle mixtures with resolved chemical and mechanical properties. Overall, these studies demonstrate that analysis of the mechanical behavior of (hydrogel) particles as well as the design of particles with controlled deformability has become an important field of research for the use of dispersed particles or particle assemblies in biomedical applications, *e*.*g*., tissue engineering.

## Supporting information

S1 Fig Height profiles of non-degraded and partially degraded microgels pOEGMA_80/0_. After certain incubation periods the particle suspension was centrifuged onto amine-functionalized glass slides. Particles were imaged by AFM in the dried state. The control refers to the height profile of non-degraded intact particles.
(PDF)

S2 Fig Double logarithmic plots of AFM force-deformation curves. Double logarithmic plots of AFM force-deformation curves obtained at the particle center and periphery of microgel particles prepared at 80°C containing 5 mol-% PEG-DA (pOEGMA_80/5_) (a) and 0 mol-% PEG-DA (pOEGMA_80/0_) (b). The solid lines represent linear fits to the linear regions of the curves. The slopes of the fits were calculated to 2.03, 2.01, 2.32 and 2.42 and are close to the theoretical value of 2.0 as predicted by the Hertzian power law for conical indenters (Eq 2).
(PDF)

S3 Fig Dependence of Young´s modulus on the radial position within the microgel. Averaged Young´s modulus along the radial position starting at the particle center of PEG-DA cross-linked microgels pOEGMA_80/5_ and cross-linker-free particles pOEGMA_80/0_. For each microgel type, several particles have been analyzed.
(PDF)

S4 Fig **Pressure-dependence of the pulse duration (a) and the magnitude of the resistive pulses (b) obtained from RPS experiments of microgels cross-linked in presence of varying amounts of PEG-DA.** Carboxylated PS particles with a mean diameter of 212 nm were used as standard and experimental data points of these particles are shown for comparison.
(PDF)

S5 Fig Depencence of particle deposition on centrifugation speed and microgel concentration. AFM height retraces in air 10 mol-% PEG-DA cross-linked microgels pOEGMA_80/10_ deposited at 20°C and different centrifugation speeds on amine-functionalized glass cover slips. From left to right: 500, 1000, 2000, and 3700 rpm (a). AFM height retraces of 10 mol-% PEG-DA cross-linked particles pOEGMA_80/10_ deposited from suspensions differing in the microgel concentration. From left to right: 0.001, 0.01, 0.1, and 0.25 mg/mL (b).
(PDF)
