# Supplementary material for: Oligo(ethylene glycol)-sidechain microgels prepared in absence of cross-linking agent: Polymerization, characterization and variation of particle deformability
Source: PLoS One. 2017 Jul 18;12(7):e0181369. doi: 10.1371/journal.pone.0181369 (PMC5515440; doi:10.1371/journal.pone.0181369)
Supplement: S1 Fig — After certain incubation periods the particle suspension was centrifuged onto amine-functionalized glass slides. Particles were imaged by AFM in the dried state. The control refers to the height profile of non-degraded intact particles. (PDF) [file pone.0181369.s001.pdf]

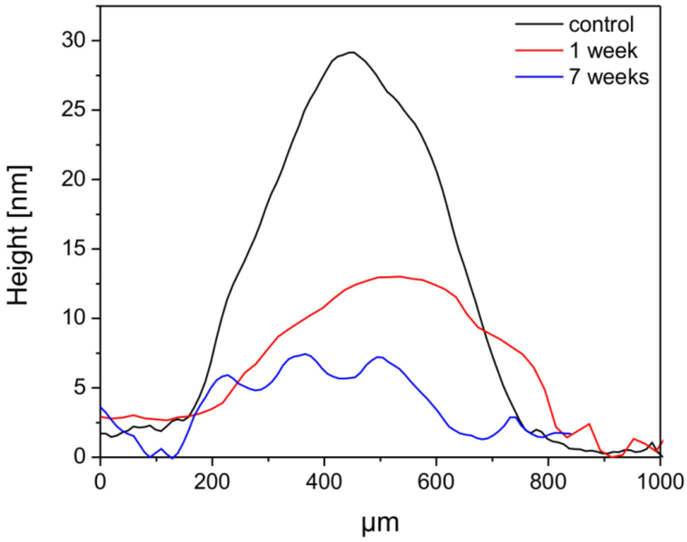

S1 Fig. Height profiles of non-degraded and partially degraded microgels pOEGMA$_{80/0}$. After certain incubation periods the particle suspension was centrifuged onto amine-functionalized glass slides. Particles were imaged by AFM in the dried state. The control refers to the height profile of non-degraded intact particles.
