# Supplementary material for: Oligo(ethylene glycol)-sidechain microgels prepared in absence of cross-linking agent: Polymerization, characterization and variation of particle deformability
Source: PLoS One. 2017 Jul 18;12(7):e0181369. doi: 10.1371/journal.pone.0181369 (PMC5515440; doi:10.1371/journal.pone.0181369)
Supplement: S2 Fig — Double logarithmic plots of AFM force-deformation curves obtained at the particle center and periphery of microgel particles prepared at 80°C containing 5 mol-% PEG-DA (pOEGMA80/5) (a) and 0 mol-% PEG-DA (pOEGMA80/0) (b). The solid lines represent linear fits to the linear regions of the curves. The slopes of the fits were calculated to 2.03, 2.01, 2.32 and 2.42 and are close to the theoretical value of 2.0 as predicted by the Hertzian power law for conical indenters (Eq 2). (PDF) [file pone.0181369.s002.pdf]

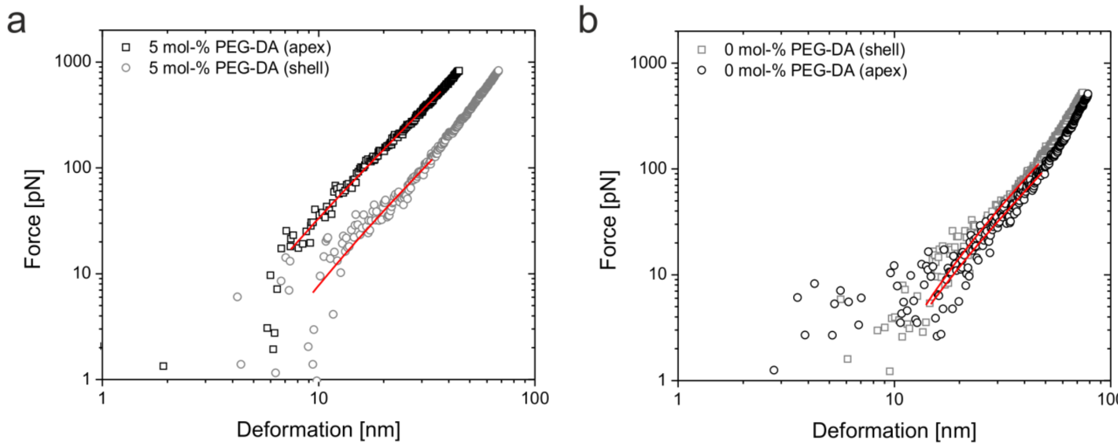

S2 Fig. Double logarithmic plots of AFM force-deformation curves. Double logarithmic plots of AFM force-deformation curves obtained at the particle center and periphery of microgel particles prepared at 80 °C containing 5 mol-% PEG-DA ($pOEGMA_{80/5}$) (a) and 0 mol-% PEG-DA ($pOEGMA_{80/0}$) (b). The solid lines represent linear fits to the linear regions of the curves. The slopes of the fits were calculated to 2.03, 2.01, 2.32 and 2.42 and are close to the theoretical value of 2.0 as predicted by the Hertzian power law for conical indenters (Eq. 2).
