# Supplementary material for: Oligo(ethylene glycol)-sidechain microgels prepared in absence of cross-linking agent: Polymerization, characterization and variation of particle deformability
Source: PLoS One. 2017 Jul 18;12(7):e0181369. doi: 10.1371/journal.pone.0181369 (PMC5515440; doi:10.1371/journal.pone.0181369)
Supplement: S3 Fig — Averaged Young´s modulus along the radial position starting at the particle center of PEG-DA cross-linked microgels pOEGMA80/5 and cross-linker-free particles pOEGMA80/0. For each microgel type, several particles have been analyzed. (PDF) [file pone.0181369.s003.pdf]

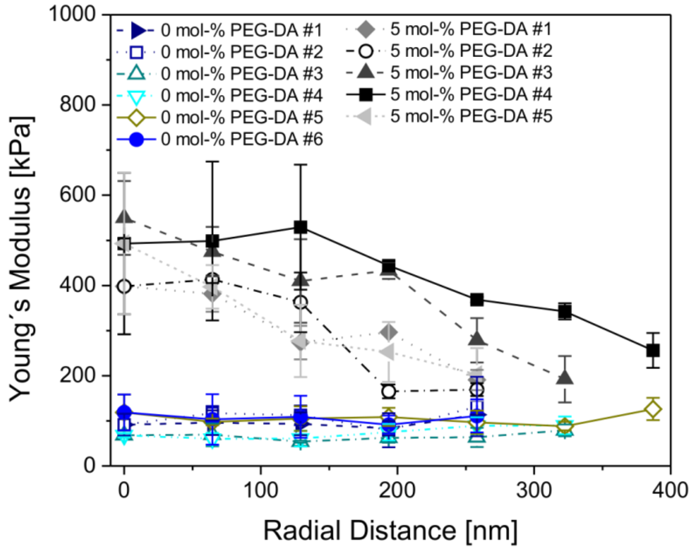

S3 Fig. Dependence of Young´s modulus on the radial position within microgel. Averaged Young´s modulus along the radial position starting at the particle center of PEG-DA cross-linked microgels $pOEGMA_{80/5}$ and cross-linker-free particles $pOEGMA_{80/0}$. For each microgel type, several particles have been analyzed.
