# Supplementary material for: Oligo(ethylene glycol)-sidechain microgels prepared in absence of cross-linking agent: Polymerization, characterization and variation of particle deformability
Source: PLoS One. 2017 Jul 18;12(7):e0181369. doi: 10.1371/journal.pone.0181369 (PMC5515440; doi:10.1371/journal.pone.0181369)
Supplement: S4 Fig — Pressure-dependence of the pulse duration (a) and the magnitude of the resistive pulses (b) obtained from RPS experiments of microgels cross-linked in presence of varying amounts of PEG-DA. Carboxylated PS particles with a mean diameter of 212 nm were used as standard and experimental data points of these particles are shown for comparison. (PDF) [file pone.0181369.s004.pdf]

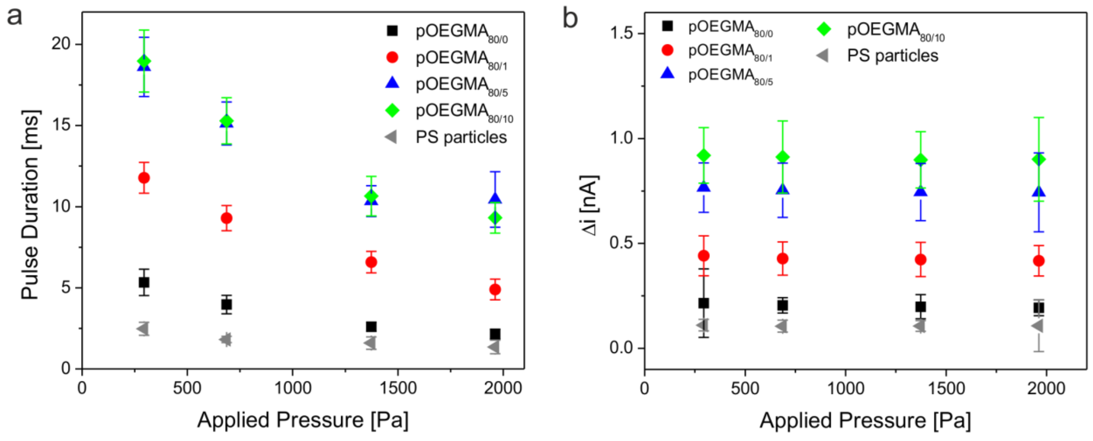


**S4 Fig. Pressure-dependence of the pulse duration (a) and the magnitude of the resistive pulses (b) obtained from RPS experiments of microgels cross-linked in presence of varying amounts of PEG-DA.** Carboxylated PS particles with a mean diameter of 212 nm were used as standard and experimental data points of these particles are shown for comparison.
