# Supplementary material for: Oligo(ethylene glycol)-sidechain microgels prepared in absence of cross-linking agent: Polymerization, characterization and variation of particle deformability
Source: PLoS One. 2017 Jul 18;12(7):e0181369. doi: 10.1371/journal.pone.0181369 (PMC5515440; doi:10.1371/journal.pone.0181369)
Supplement: S5 Fig — AFM height retraces in air 10 mol-% PEG-DA cross-linked microgels pOEGMA80/10 deposited at 20°C and different centrifugation speeds on amine-functionalized glass cover slips. From left to right: 500, 1000, 2000, and 3700 rpm (a). AFM height retraces of 10 mol-% PEG-DA cross-linked particles pOEGMA80/10 deposited from suspensions differing in the microgel concentration. From left to right: 0.001, 0.01, 0.1, and 0.25 mg/mL (b). (PDF) [file pone.0181369.s005.pdf]

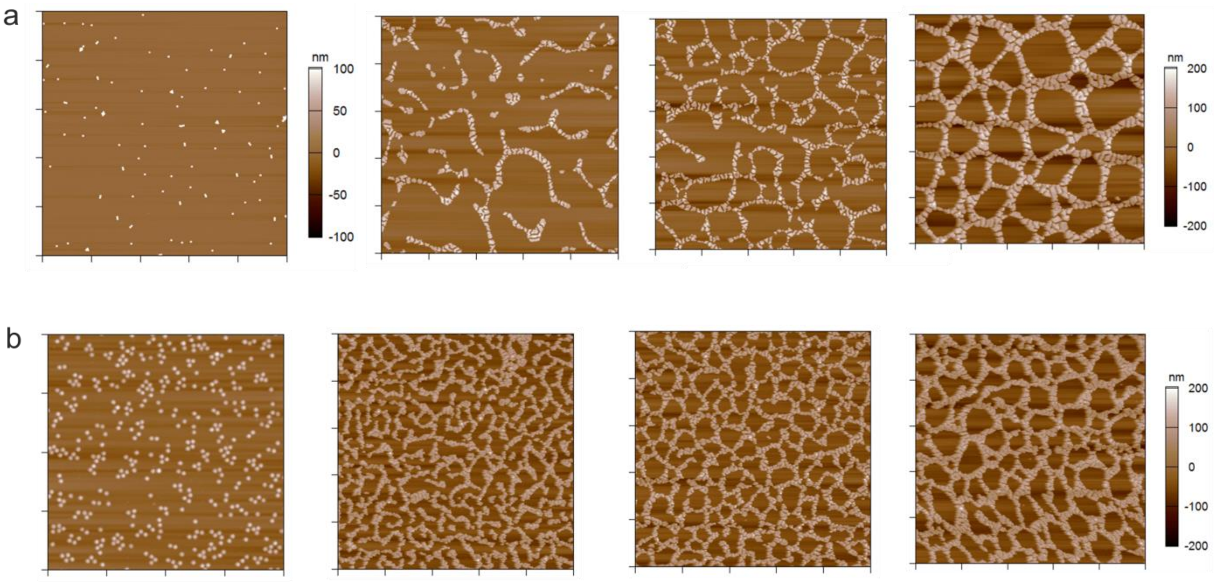


**S5 Fig. Depencence of particle deposition on centrifugation speed and microgel concentration.** AFM height retraces in air 10 mol-% PEG-DA cross-linked microgels pOEGMA$_{80/10}$ deposited at 20 °C and different centrifugation speeds on amine-functionalized glass cover slips. From left to right: 500, 1000, 2000, and 3700 rpm (a). AFM height retraces of 10 mol-% PEG-DA cross-linked particles pOEGMA$_{80/10}$ deposited from suspensions differing in the microgel concentration. From left to right: 0.001, 0.01, 0.1, and 0.25 mg/mL (b).
